# Were cancer patients worse off than the general population during the COVID-19 pandemic? A population-based study from Norway, Denmark and Iceland during the pre-vaccination era

**DOI:** 10.1016/j.lanepe.2023.100680

**Published:** 2023-07-10

**Authors:** Anna L.V. Johansson, Anna Skog, Tom Børge Johannesen, Tor Åge Myklebust, Charlotte Wessel Skovlund, Lina Steinrud Mørch, Søren Friis, Mads Gamborg, Marnar Fríðheim Kristiansen, David Pettersson, Elínborg J. Ólafsdóttir, Helgi Birgisson, Runolfur Palsson, Elias Eythorsson, Sandra Irenaeus, Mats Lambe, Giske Ursin

**Affiliations:** a Cancer Registry of Norway, P.O. Box 5313 Majorstuen, Oslo N-0304, Norway; b Department of Medical Epidemiology and Biostatistics, Karolinska Institutet, P.O. Box 281, Stockholm SE-17177, Sweden; c Department of Research and Innovation, Møre and Romsdal Hospital Trust, Ålesund, Norway; d Cancer Surveillance and Pharmacoepidemiology, Danish Cancer Society Research Center, Danish Cancer Society, Strandboulevarden 49, Copenhagen DK-2100, Denmark; e Center of Health Science, Faculty of Health Sciences, Tórshavn, Faroe Islands; f National Hospital of the Faroe Islands, Tórshavn, Faroe Islands; g National Board of Health and Welfare, Stockholm SE-106 30, Sweden; h ICS Research and Registration Center, Icelandic Cancer Society, P.O. Box 5420, 105 Reykjavík, Iceland; i Landspitali-The National University Hospital of Iceland, Saemundargata 2, 102 Reykjavík, Iceland; j University of Iceland, Reykjavik, Iceland; k Regional Cancer Center Central Sweden, Akademiska Sjukhuset, Uppsala SE-751 85, Sweden; l Department of Immunology, Genetics and Pathology, Uppsala University, Uppsala, Sweden; m Institute of Basic Medical Sciences, University of Oslo, Oslo, Norway; n Department of Preventive Medicine, University of Southern California, Los Angeles, CA, USA

**Keywords:** COVID-19, Coronavirus disease 2019, SARS-CoV-2, Severe acute respiratory syndrome coronavirus 2, Cancer, Hospitalisation, Critical care outcomes, Intensive care, Mortality, Nordic, Denmark, Norway, Iceland

## Abstract

**Background:**

In a population-based setting, we investigated the risks of testing positive for SARS-CoV-2 and developing severe COVID-19 outcomes among cancer patients compared with the general population.

**Methods:**

In nationwide cohorts, we identified all individuals in Norway, Denmark and Iceland who tested positive for SARS-CoV-2 or had a severe COVID-19 outcome (hospitalisation, intensive care, and death) from March until December 2020, using data from national health registries. We estimated standardised incidence ratios (SIRs) with 95% confidence intervals (CIs) comparing cancer patients with the general population.

**Findings:**

During the first wave of the pandemic, cancer patients in Norway and Denmark had higher risks of testing SARS-CoV-2 positive compared to the general population. Throughout 2020, recently treated cancer patients were more likely to test SARS-CoV-2 positive. In Iceland, cancer patients experienced no increased risk of testing positive. The risk of COVID-19-related hospitalisation was higher among cancer patients diagnosed within one year of hospitalisation (Norway: SIR = 2.43, 95% CI 1.89–3.09; Denmark: 2.23, 1.96–2.54) and within five years (Norway: 1.58, 1.35–1.83; Denmark: 1.54, 1.42–1.66). Risks were higher in recently treated cancer patients and in those diagnosed with haematologic malignancies, colorectal or lung cancer. Risks of COVID-19-related intensive care and death were higher among cancer patients.

**Interpretation:**

Cancer patients were at increased risk of testing positive for SARS-CoV-2 during the first pandemic wave when testing availability was limited, while relative risks of severe COVID-19 outcomes remained increased in cancer patients throughout 2020. Recent cancer treatment and haematologic malignancy were the strongest risk factors.

**Funding:**

Nordic Cancer Union.

**Research in context**

**Evidence before this study**

Early in the COVID-19 pandemic, cancer patients were identified as a high-risk group for severe COVID-19 outcomes. Some evidence, largely based on clinical cohorts, suggested that haematologic malignancies and recent treatment were associated with higher risks, although population-based data are lacking. There is also limited evidence on the risk of testing positive for SARS-CoV-2 in cancer patients. We searched PubMed from database inception until December 2022, for published articles in English on the risk of testing positive for SARS-CoV-2 and severe COVID-19 outcomes in patients with cancer, using the search terms (“COVID-19” OR “SARS-CoV-2”) AND (“cancer” OR “malignancy”) AND (“incidence” OR “risk” OR “testing” OR “severe outcome” OR “hospitalisation” OR “intensive care” OR “death”).

**Added value of this study**

In a population-based setting including three countries, we utilised harmonised data from individually linked cancer registries to patient registries, pandemic registries, and cause of death information covering a study period from March to December, 2020. We found that patients undergoing recent therapy and those diagnosed with haematologic malignancies were at increased risk of testing positive for SARS-CoV2 compared with the general population, in particular during the first wave of the pandemic. Furthermore, we found that patients who had undergone recent cancer therapy were at higher risk of severe COVID-19 outcomes compared with the general population, a/s were patients diagnosed with haematologic malignancies, lung cancer, and colorectal cancer.

**Implications of all the available evidence**

The results suggest that cancer patients were worse off than the general population, in particular with respect to severe COVID-19 outcomes. These findings are generalisable to the pre-vaccination era during 2020, and may indicate which patient groups should be carefully monitored in the post-vaccination era.

## Introduction

The COVID-19 pandemic has had a severe impact on global health, especially in 2020 during the first year of the pandemic before vaccines were available. In Norway, Denmark, and Iceland, the incidence rates of COVID-19 peaked during a first wave between March and May and again during a second wave between October and December (Table 1, Supplementary Figure S1). The second peak occurred earlier in Iceland and was highest in Denmark.

**Table 1:** The incidence of SARS-CoV-2 positive testing and severe COVID-19-related outcomes in cancer patients and the general population during March–December 2020 (men and women aged ≥18 years).

	Norway	Denmark	Iceland
	N (per 100,000)	N (per 100,000)	N (per 100,000)
**General population**^a^	4,263,169	4,674,589	284,563
SARS-CoV-2 positive	41,468 (972.7)	166,522 (3562.3)	4094 (1438.7)
COVID-19 hospitalisation	2361 (55.4)	8230 (176.1)	285 (100.2)
COVID-19 ICU admission	379 (8.9)	911 (19.5)	49 (17.2)
COVID-19 ICU mechanical ventilation	293 (6.9)	577 (12.3)	25 (8.8)
COVID-19 death	407 (9.5)	1019 (21.8)	28 (9.8)
**Cancer diagnosed <5 years before testing positive for SARS-CoV-2**			
SARS-CoV-2 positive	730	2758	72
COVID-19 hospitalisation	174	652	22
COVID-19 ICU admission	38	76	3
COVID-19 ICU mechanical ventilation	28	56	1
COVID-19 death	40	108	1
**Cancer diagnosed <1 year before testing positive for SARS-CoV-2**			
SARS-CoV-2 positive	203	727	19
COVID-19 hospitalisation	67	241	6
COVID-19 ICU admission	13	35	1
COVID-19 ICU mechanical ventilation	7	27	0
COVID-19 death	12	44	0

a Includes men and women aged 18 or above.

During the first wave of the pandemic, reports emerged suggesting that male sex, old age, and comorbid conditions, including cancer, were associated with a higher risk of contracting SARS-CoV-2 and experiencing severe COVID-19-related outcomes such as hospitalisation and death.^1^ The limited availability of testing during the early phase hampered the interpretation of some of the early reports, as testing was prioritised in patients who were admitted to hospital and healthcare staff, which may have introduced bias.^2^

Later in the pandemic, some studies suggested that patients with cancer experienced more severe COVID-19 disease, in particular those recently treated for cancer or those who had haematologic malignancies.^3^, ^4^, ^5^, ^6^, ^7^, ^8^ However, patients with prevalent cancer represent a heterogeneous group. Accordingly, different COVID-19-related risks have been estimated for cancer type, recent therapy, and time since cancer diagnosis.

Using the powerful and linkable population-based health registries in Norway, Denmark and Iceland, we aimed to determine whether cancer patients had higher risks of testing positive for SARS-CoV-2 and developing severe COVID-19-related outcomes (hospitalisation, intensive care unit [ICU] admission including mechanical ventilation, and death) from March to December 2020. We assessed whether cancer type, time since cancer diagnosis, treatment modality, and recent cancer treatment influenced the risk of testing positive for SARS-CoV-2 and developing severe COVID-19 outcomes. We also assessed potential interactions by analyzing these associations across sex, age groups, and the first and second waves of the pandemic.

## Methods

### Study population

This population-based study included nationwide health registry data from Norway, Denmark, and Iceland. In each country, the national cancer registry was linked to other national registries, including patient registries, infectious disease registry and/or pandemic registry, and cause of death registry to obtain information on cancer, positive SARS-CoV-2 tests and severe COVID-19 outcomes (Supplementary Table S1). Reporting to the national health registries from the health services is mandatory by law, and thus the completeness and validity are high.^9^ The study population consisted of the entire populations aged 18 years or older in each country (Norway N = 4,263,169, Denmark N = 4,674,589, Iceland N = 284,563) that were followed for a positive test of SARS-CoV-2 and COVID-19-related outcomes during March 1–December 31, 2020 (Table 1). We only counted the first positive SARS-CoV-2 test per person, since re-infection rates were low during 2020. All countries were included in the analysis of the risk of SARS-CoV-2 positive test, while the analysis of severe COVID-19 outcomes was restricted to Norway and Denmark due to small numbers of severe COVID-19 outcomes in Iceland (Table 1).

### Definitions of COVID-19 outcomes

A positive test of SARS-CoV-2 was defined as a positive laboratory confirmed test recorded in a COVID-19 registry or a record of a COVID-19 diagnosis using the 10th version of the International Classification of Diseases (ICD-10), code U07.1 (Supplementary Table S2). Hospitalisation due to COVID-19 was defined as either a) hospital admission with primary discharge diagnosis of COVID-19 (ICD-10 code U07.1), or b) hospital admission with a previous positive SARS-CoV-2 test within 14 days. Intensive care admission due to COVID-19 was defined as admission to an ICU with a primary discharge diagnosis of COVID-19. Mechanical ventilation during intensive care admission due to COVID-19 was defined as a) ventilator with intubation and invasive positive pressure ventilation or b) other breathing support, including non-invasive ventilation (in Denmark). Death related to COVID-19 was defined as a) death with COVID-19 as underlying cause of death, or b) death within 30 days after admission to hospital with COVID-19 as primary discharge diagnosis. The dates of each COVID-19 outcome were recorded as exact dates (in Norway, year and month).

### Definition of cancer status and treatments

Cancer diagnoses from each national cancer registry were included with date of diagnosis and type of tumour according to ICD-10. We included all cancer types (“all sites”) except non-melanoma skin cancer (ICD-10 codes C00–C96, excluding C44; also including D32, D33, D42, D43, D35.3, D35.4, D44.3, D44.4, D44.5, D45, D46, D47) (Supplementary Table S2). Major cancer sites were analysed separately and included breast (C50), prostate (C61), melanoma (C43), colorectal (C18–C20), lung (C33–C34), and haematologic malignancies (C81–C96, D45–D47). Cancer exposure status was defined as a recorded cancer diagnosis within one year (0–12 months) or five years (0–60 months) prior to the first day of the month of each COVID-19 outcome. For each month of 2020, a moving exposure window (12 or 60 months) was applied to count exposed COVID-19 cases prior to each month of 2020.

Cancer therapy was classified as chemotherapy, radiotherapy, immunotherapy, and targeted therapy as recorded by procedure codes and anatomical therapeutic chemical (ATC) codes in the patient and prescribed drugs registries (Supplementary Table S2). For Denmark, cancer therapy was classified according to diagnosis-related groups (DRG).^10^ A team of co-authors (TBJ, SI, SF) assessed comparability of codes across the three study countries to approach harmonisation of definitions.

### Statistical methods

Standardised incidence ratios (SIRs) were estimated as observed over expected numbers of positive SARS-CoV-2 tests and severe COVID-19 outcomes in persons with cancer within one and five years per month in 2020. The observed numbers were calculated within population strata of age (Norway, Denmark: five year intervals; Iceland: 0–69, 70+), sex (male, female), and bimonthly intervals 2020 (March–April, May–June, July–August, September–October, November–December). The expected numbers were calculated as the number of person-years at risk in the cancer patients multiplied with the expected outcome rate in the general population within each age-sex-month stratum. The expected outcome rates were estimated by the number of COVID-19 outcomes per month divided by person-years at risk in the general population. General population counts were obtained as mid-population counts from each country’s national statistics office (in Denmark, exact population counts per quarter of year were available), where each person contributed 1/12 of a person-year to each month in the SIR estimation. For each COVID-19 outcome, SIRs by cancer status (cancer within one or five years) were estimated with exact 95% confidence intervals (CI) based on the Poisson distribution to account for sparse data.^11^^,^^12^ Furthermore, SIRs by cancer status were calculated within levels of age, sex, month, cancer type, and treatment modality, corresponding to interaction effects between cancer status and each variable.

In a sensitivity analysis based on the Norwegian data, we used an alternative analytic approach for severe COVID-19 outcomes restricted to persons who tested positive for SARS-CoV-2, which represent the most common design for studies of COVID-19 outcomes. We assessed severe COVID-19 outcomes within 30 days of the positive COVID-19 test as binary variables, which captured >95% of all severe COVID-19 outcomes in the cohort. Odds ratios (OR) with 95% CIs for the association between cancer status and severe outcomes were estimated using logistic regression assuming complete 30-day follow-up of cohort members testing positive. The ORs were interpreted as relative risks based on the rare disease assumption.^13^ Relative risks (RR) with 95% CIs were also estimated using Poisson regression to obtain a direct estimate of the relative risk, which does not rely on the rare disease assumption. The regression models were adjusted for age, sex, month, and region to obtain the main effect of cancer and therapy on risk of developing severe COVID-19 outcomes. As the sensitivity analysis was performed among COVID-19 positive patients, it may have depended on testing strategies during the pandemic, with cancer patients being more or less likely to be tested during different months of 2020. All analyses were performed with Stata 17/MP and SAS version 9.4. The significance level was 5% and tests were two-sided.

### Role of the funding source

The sponsor and the funder of the study had no role in study design, data collection, data analysis, data interpretation, or writing of the report.

## Results

### Risk of testing positive for SARS-CoV-2

During 2020, 727 (Denmark), 203 (Norway), and 19 (Iceland) patients with recently (within one year) diagnosed cancer tested positive for SARS-CoV-2, while 2758 (Denmark), 730 (Norway), and 72 (Iceland) tested positive within five years after their cancer diagnosis (Table 1).

**Fig. 1:**
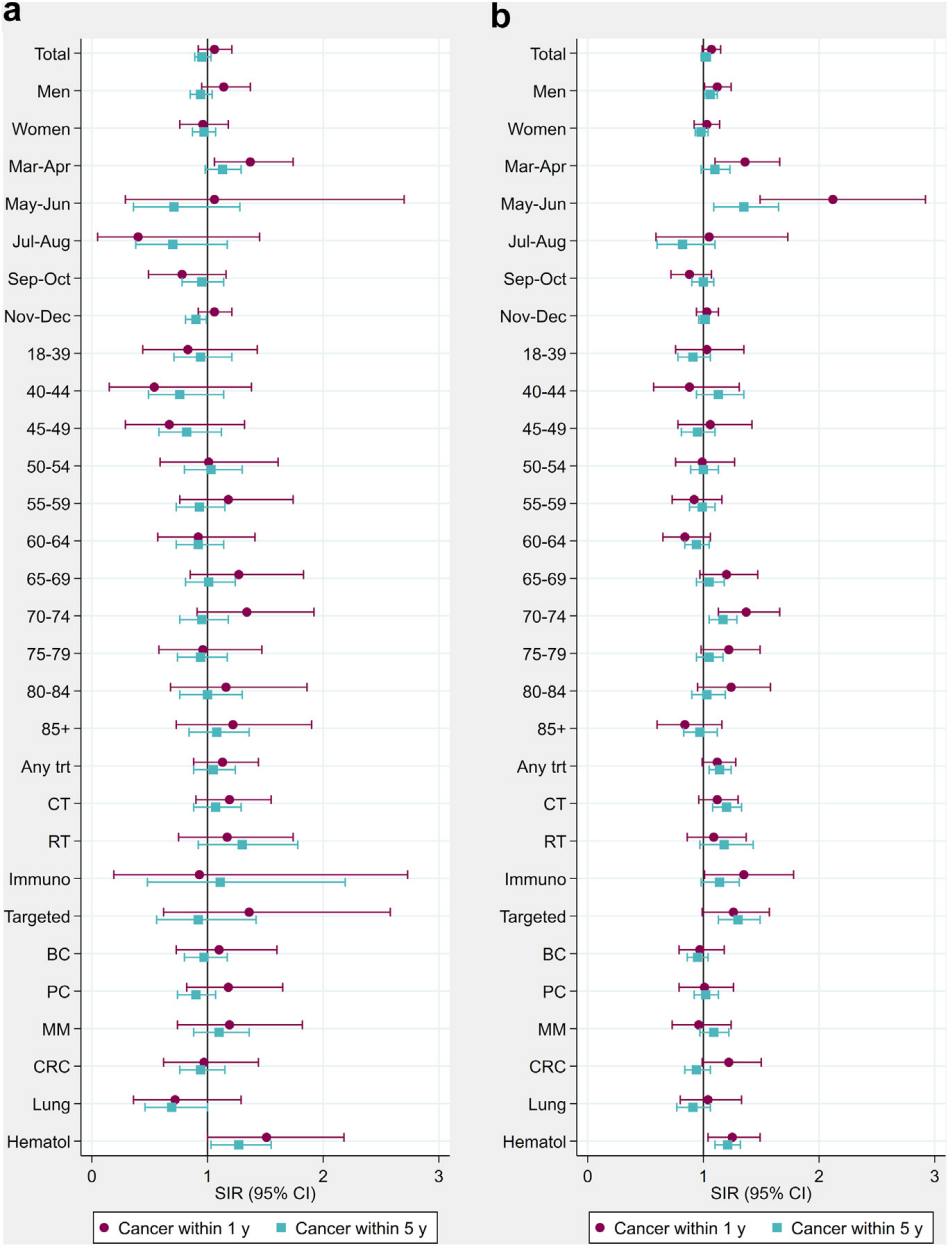
**Standardised incidence ratios (SIRs) of SARS-CoV-2 positive test in cancer patients diagnosed within 1 or 5 years from the infection by subgroups of sex, months, age, cancer therapy and cancer types, during March–December 2020 in a) Norway and b) Denmark.Estimates are presented in**Supplementary Table S3. Any trt, any treatment; CT, chemotherapy; RT, radiotherapy; Immuno, immunotherapy; Targeted, targeted therapy; BC, breast cancer; PC, prostate cancer; MM, malignant melanoma; CRC, colorectal cancer; Lung, lung cancer; Hematol, haematologic malignancies.

**Fig. 2:**
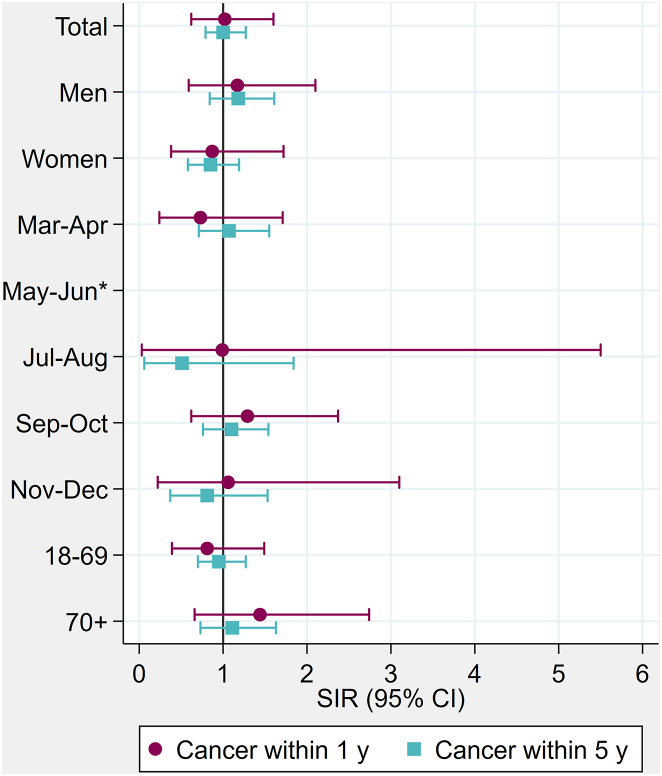
**Standardised incidence ratios (SIRs) of SARS-CoV-2 positive test in cancer patients diagnosed within 1 or 5 years from the infection by subgroups of sex, month in 2020 and age, during March–December 2020 in Iceland.Estimates are presented in**Supplementary Table S3. ∗No estimate for cancer within 1 or 5 year due to sparse data.

Compared to the general population, Danish cancer patients experienced a similar risk of testing positive for SARS-CoV-2 within one year after the diagnosis (SIR: 1.07, 95% CI 0.99–1.15), except in men (1.12, 1.01–1.24) and during the first pandemic wave (March–April: 1.36, 1.10–1.66; May–June: 2.12, 1.49–2.92) (Fig. 1, Supplementary Table S3). Having been diagnosed with cancer within five years was overall not associated with testing positive for SARS-CoV-2 compared to the general population, although male cancer patients experienced a slightly increased risk (1.06, 1.01–1.12). In Norway, cancer patients were at an increased risk of testing positive for SARS-CoV-2 within one year after diagnosis during March–April (1.36, 1.06–1.74), whereas there was no association with sex. The risk of SARS-CoV-2 positivity was not increased among Icelandic cancer patients during the first or second wave and was not affected by sex (Fig. 2, Supplementary Table S3). Age of cancer patients was not associated with risk of testing positive for SARS-CoV-2, except for Danish cancer patients aged 70–74 years (within one year after diagnosis: 1.37, 1.13–1.66).

Among cancer patients diagnosed within one year of a SARS-CoV-2 test, recent chemotherapy or radiotherapy was not associated with increased risk of positive SARS-CoV-2 test, except in Danish patients with recent immunotherapy (1.35, 1.01–1.78) or targeted therapy (1.26, 0.99–1.57) (Fig. 1, Supplementary Table S3). Also, Danish cancer patients diagnosed within five years of SARS-CoV-2 testing and with recent cancer therapy were at increased risks for testing positive (any treatment: 1.14, 1.05–1.24; chemotherapy: 1.20, 1.08–1.33; targeted therapy: 1.30, 1.13–1.49).

A diagnosis of a haematologic malignancy within one year of SARS-CoV-2 testing was associated with increased risk of testing positive among both Danish (1.25, 1.04–1.49) and Norwegian (1.51, 1.00–2.18) cancer patients, and also among cancer patients diagnosed with haematologic malignancies within five years of the test (Denmark: 1.21, 1.10–1.32; Norway: 1.27, 1.03–1.55).

### Risk of severe COVID-19 outcomes

**Fig. 3:**
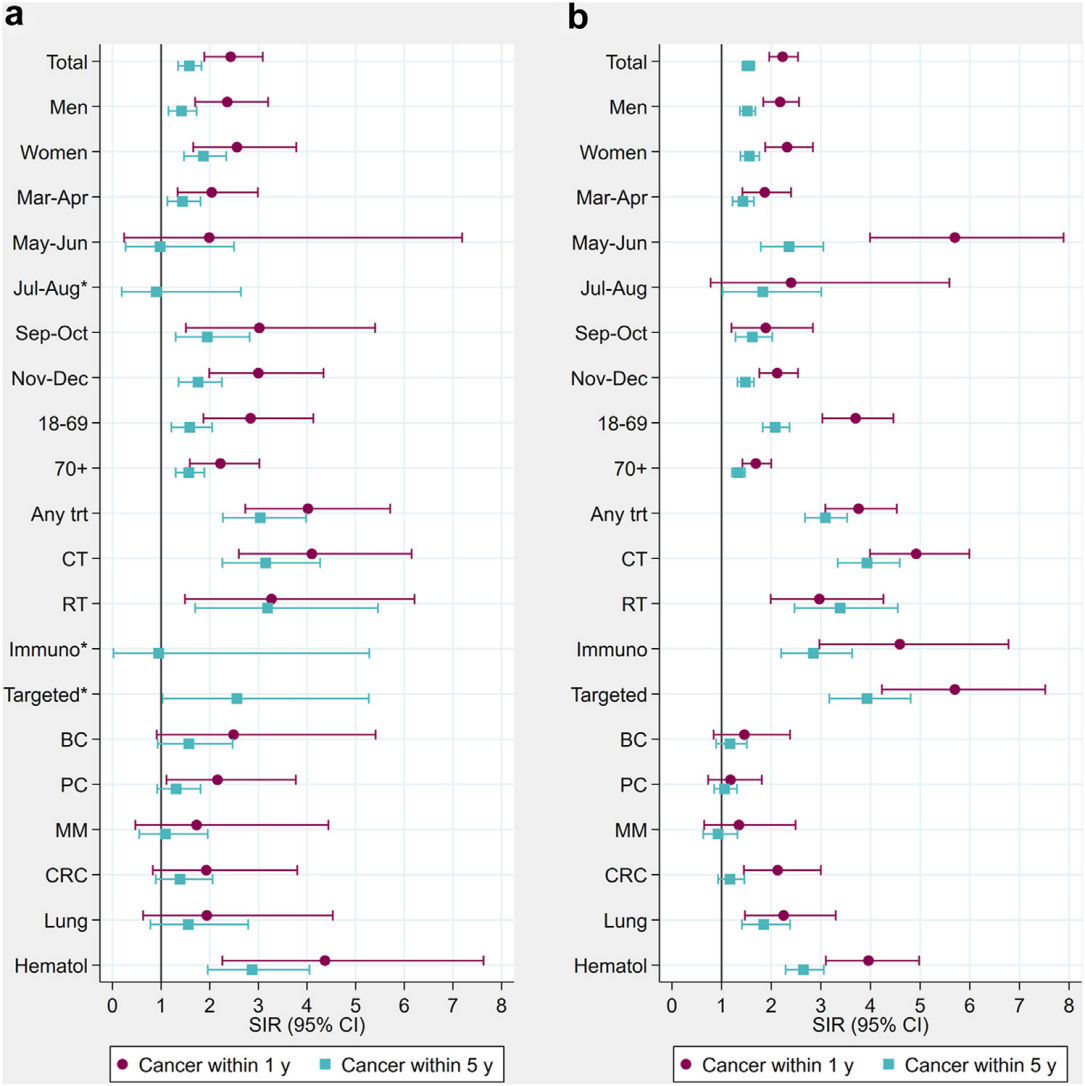
**Standardised incidence ratios (SIRs) of COVID-19-related hospitalisation in cancer patients diagnosed within 1 or 5 years from the hospitalisation by subgroups of sex, months, age, cancer therapy and cancer types, during March–December 2020 in a) Norway and b) Denmark.Estimates are presented in**Supplementary Tables S4 and S5. ∗No estimate for cancer within 1 year due to sparse data. Any trt, any treatment; CT, chemotherapy; RT, radiotherapy; Immuno, immunotherapy; Targeted, targeted therapy; BC, breast cancer; PC, prostate cancer; MM, malignant melanoma; CRC, colorectal cancer; Lung, lung cancer; Hematol, haematologic malignancies.

**Fig. 4:**
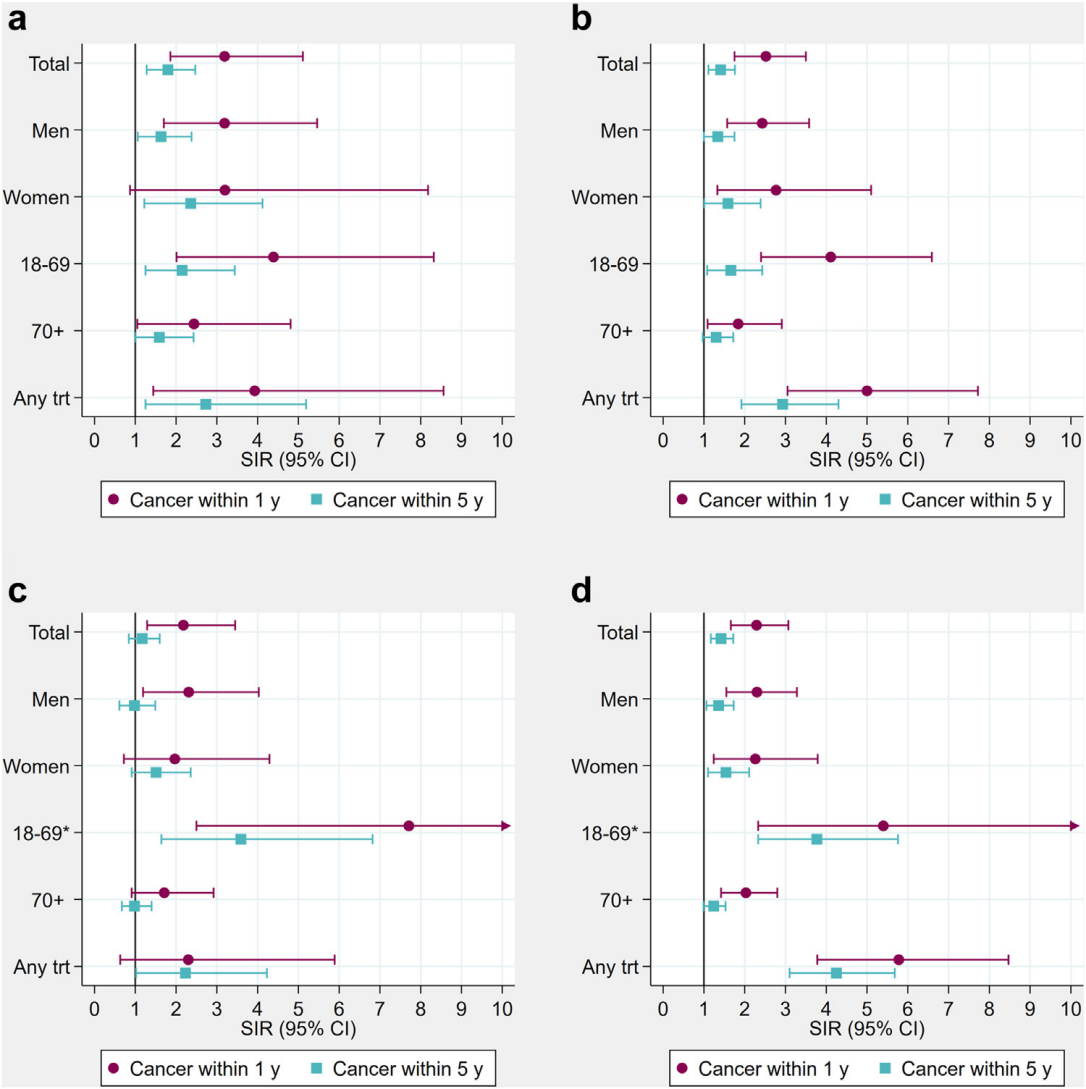
**Standardised incidence ratios (SIRs) of COVID-19-related intensive care unit admissions and COVID-19-related death in cancer patients diagnosed within 1 or 5 years from the admission or death by subgroups of sex, age and cancer therapy, during March–December 2020.Panels showing a) COVID-19-related intensive care in Norway, b) COVID-19-related intensive care in Denmark, c) COVID-19-related death in Norway, and d) COVID-19-related death in Denmark. Estimates are presented in**Supplementary Tables S4 and S5. ∗Upper confidence limit has been trimmed to value 10. Exact limits provided in Supplementary Tables S4 and S5. Any trt, any treatment.

Compared to the general population, Norwegian patients diagnosed with cancer within five years were at increased risk of COVID-19-related hospitalisation (SIR = 1.58, 95% CI 1.35–1.83), intensive care (1.80, 1.28–2.47) and mechanical ventilation (1.71, 1.14–2.47), but not of death (1.17, 0.84–1.60) (Fig. 3 panel a, Fig. 4 panel a, c). In Denmark, all severe COVID-19 outcomes were associated with cancer diagnosed within five years (hospitalisation (1.54, 1.42–1.66), intensive care (1.41, 1.11–1.76), mechanical ventilation (1.62, 1.22–2.10), and death (1.42, 1.17–1.72) (Fig. 3 panel b, Fig. 4 panel b, d). Among cancer patients diagnosed within one year, the associations were even stronger in both Norway (hospitalisation: 2.23 (1.96–2.54), intensive care: 2.52 (1.75–3.50), and death: 2.29 (1.66–3.07)) and Denmark (hospitalisation: 2.43 (1.89–3.09), intensive care: 3.19 (1.86–5.11), and death: 2.18 (1.29–3.45).

### Severe COVID-19 outcomes in subgroups by age, sex, treatment, and type of cancer

In both Denmark and Norway, the risks of hospitalisation due to COVID-19 were significantly higher in both men (NO: 2.36, 1.70–3.20; DK: 2.18, 1.84–2.56) and women (NO: 2.56, 1.66–3.78; DK: 2.32, 1.88–2.84) with cancer compared to the general population, and during both the first (March–June) and second (September–December) waves of the pandemic (Fig. 3, Supplementary Tables S4 and S5). The increased risk for hospitalisation was also present among both younger (<70) and older (≥70) patients, in particular among younger patients in Denmark (2.08, 1.83–2.37). Recent cancer treatments (any, chemotherapy, radiotherapy, immunotherapy, and targeted therapy) were strongly associated with risk of hospitalisation due to COVID-19, with the strongest association observed among patients diagnosed within one year. Having a haematologic malignancy was associated with increased risks of hospitalisation in both Norway (4.37, 2.26–7.63) and Denmark (3.96, 3.10–4.98), and in Denmark also colorectal cancer within one year and lung cancer within one and five years was associated with higher risk.

Cancer within one or five years was associated with increased risk of COVID-19-related ICU admission and death in both Norway and Denmark (Fig. 4). Associations for mechanical ventilation were of similar strengths as those for ICU admission, albeit with limited power (Supplementary Tables S4 and S5). In both countries, the associations were stronger among cancer patients <70 years of age diagnosed within one year, and among those with recent treatment (any, chemotherapy, immunotherapy, and targeted therapy). In Denmark, the risk of intensive care was increased during the first pandemic wave (March–June), while the risk of death was increased during both waves (May–June and September–December) (Supplementary Table S5). Haematologic malignancies were strongly associated with COVID-19-related ICU admission (NO: 7.64, 2.08–19.56; DK: 5.93, 3.24–9.94) and death (NO: 5.64, 1.83–13.16; DK: 4.15, 2.32–6.85) in both Norway and Denmark, as was colorectal cancer within one year and lung cancer within one and five years in Denmark. No associations were observed with other cancer types.

### Sensitivity analysis

To evaluate the influence of the estimation method, we estimated odds ratios and relative risks for severe COVID-19 outcomes among COVID-19 positive patients in Norway. For COVID-19-related hospitalisation, the SIR estimate was lower than the OR yet similar to the RR (Supplementary Figure S2). Higher ORs for more common outcomes indicates a violation of the rare disease assumption for the interpretation of the OR as a relative risk. For the rarer outcomes (ICU, mechanical ventilation, and death), the three measures SIR, OR, and RR were similar, as one would expect if the rare disease assumption holds.

## Discussion

In this population-based multi-country study covering 9 million adults, we found a slightly increased risk of testing positive for SARS-CoV-2 in recently diagnosed cancer patients during the first wave (March–June 2020) of the pandemic in Norway and Denmark, but not during the second wave (September–December). The higher risk of testing positive was consistently associated with haematologic malignancy, slightly associated with male sex, but not with age. Danish cancer patients who had recently undergone cancer therapy also experienced an increased risk of testing positive for SARS-CoV-2. The risk of testing positive for SARS-CoV-2 was not increased among Icelandic cancer patients during the first or second waves of COVID-19.

The risks of severe COVID-19 outcomes were consistently increased in cancer patients during the first and second waves, in both men and women, across all ages, and among patients below 70 years. Recent cancer therapy, including chemotherapy, radiotherapy, immunotherapy, and targeted therapy, was strongly associated with higher risks of hospitalisation, intensive care admission, and death. Haematologic malignancies, lung, and colorectal cancers were associated with increased risks of severe COVID-19 outcomes, in particular intensive care admission and death. The associations for the severe COVID-19 outcomes were strongest among Danish cancer patients, while associations among Norwegian cancer patients were often non-significant, likely due to lower rates of COVID-19 in the Norwegian population.

Only a few studies have assessed the risk of testing positive for SARS-CoV-2 in cancer patients, and with some suggesting a slightly higher risk for patients with inactive cancer,^6^^,^^14^ but only one study reported higher risks for patients with active cancer,^8^ and two suggested a lower risk.^15^^,^^16^ In line with our findings, Roel et al. and Serraino et al. reported increased risks among patients with haematologic malignancies.^8^^,^^14^ However, in contrast to our results, the authors also found an association with high age, but not by first and second wave of 2020.^8^^,^^14^ We found an increased risk of testing positive for SARS-CoV-2 during the first wave of the pandemic in Norway and Denmark. This association was not seen in Iceland despite the similarities in COVID-19 incidence and the progression of the pandemic in all three countries (Supplementary Figure S1).

Several comprehensive meta-analyses have been published on severe COVID-19 outcomes in cancer patients.^9^^,^^17^, ^18^, ^19^, ^20^, ^21^ There is consistent evidence that cancer patients are at an increased risk for severe COVID-19 outcomes, with particularly high risks in patients undergoing chemotherapy and with haematologic malignancies.^4^, ^5^, ^6^, ^7^^,^^21^, ^22^, ^23^ Patients with haematologic malignancies may have a reduced immune response to SARS-CoV-2, which may partly be explained by the underlying malignancy, or the cancer treatment.^24^ Associations are weaker for solid tumors, and one meta-analysis found no association between cancer therapy and COVID-19-related death.^25^ In line with some,^4^^,^^26^ but not all studies,^18^^,^^19^ we found an association with immunotherapy and severe COVID-19 outcomes. Lazarus et al. reported an increased risk for COVID-19-related death only in patients with previous chemoimmunotherapy,^18^ while another study reported an increased risk for cancer patients undergoing targeted therapy.^15^ There is also growing evidence that lung cancer is associated with higher risks of severe COVID-19 outcomes,^7^^,^^8^^,^^16^^,^^17^^,^^26^ while no other study to our knowledge has reported an increased risk in patients with colorectal cancer. Possible explanations for the higher risk of severe COVID-19 outcomes in cancer patients include altered clinical evolution of COVID-19 caused by the cancer treatment, increased risk of secondary infections due to cancer-related immune suppression, and higher risk of thromboembolic events associated with increased mortality in cancer patients.^24^^,^^27^ In addition, cancer patients receiving treatment may have more advanced disease, which may increase their risk of severe COVID-19 outcomes, though they may also be healthier and more capable of tolerating treatment compared with other cancer patients, in particular older and those with high comorbidity.

We found similar risks of severe COVID-19 outcomes among men and women. This is in contrast to earlier studies suggesting that male cancer patients have higher risks of severe COVID-19 outcomes than female cancer patients.^14^ There are also reports of increased risks in both young and old patients.^4^^,^^14^^,^^17^ One previous study found,^14^ in line with our results, increased risks both during the first and second wave during 2020, while other studies have reported increased risks only during the first wave or the second wave.^27^^,^^28^

Early in the pandemic, cancer patients were identified as a high-risk group for testing positive for SARS-CoV-2 and developing severe COVID-19 outcomes, particularly those undergoing active therapy and with haematologic malignancies. Since hospital patients were prioritised for SARS-CoV-2 testing in the early phase of the pandemic in Denmark and Norway due to limited testing capacity, this could have led to biased associations in the earlier studies. In the latter half of 2020, the occurrence rates of severe COVID-19 outcomes increased, a finding which can likely be explained by the development of the pandemic as well as the increased availability of diagnostic tests. Importantly, although cancer patients experienced increased relative risk for severe COVID-19 outcomes, the absolute risks for these outcomes were low and cancer patients only accounted for a small proportion of all patients with severe COVID-19 outcomes. It is also noteworthy that the increased risks for severe COVID-19 outcomes in cancer patients remained during the second COVID-19 wave in 2020, when COVID-19 therapy had improved.

Our findings are generalisable to the pre-vaccination era of the COVID-19 pandemic. Recent studies have indicated that although vaccination protects against severe COVID-19, the higher risk among cancer patients may still be present after vaccination,^28^ in particular among patients with haematologic malignancies.^29^ Systematic monitoring of severe COVID-19 outcomes among cancer patients is consequently imperative.

This study is one of the largest to date assessing COVID-19-related risks and severe outcomes among cancer patients by including high-quality nationwide registry data from the Nordic region. Via individual-level record linkages, we established a population-based cohort of cancer patients with essentially complete information on cancer diagnoses, positive SARS-CoV-2 tests and severe COVID-19 outcomes, which was then compared to rates of positive tests and severe COVID-19 outcomes in the general population. This ensured valid and unbiased estimation of effects and associations, especially since we compared cancer patients with a non-cancer population without conditioning on testing positive, which is sometimes lacking in other studies. In the analysis of severe COVID-19 outcomes, we included a series of nested outcomes, ranging from hospitalisation to death, in order to elucidate the influence of cancer throughout the disease process. An important strength was the use of SIRs, which compared the risks among cancer patients in comparison to the general population, both overall and within subgroups of age, sex, cancer types, treatments, and monthly intervals. Such comparisons correspond to interaction effects between cancer status and each subgroup factor, which may elucidate the independent effect of cancer within each subgroup. Our sensitivity analysis, which similarly to other studies conditioned on testing positive, indicated that the use of logistic regression for common outcomes, such as hospitalisation due to COVID-19, might be prone to violation of the rare disease assumption required for odds ratios to be valid estimates of the corresponding relative risks. In this regard, the agreement between the SIRs, odds ratios and risk ratios for severe COVID-19 outcomes (ICU, mechanical ventilation, and death) indicated robustness of the reported outcomes in our study. Finally, we had complete and detailed information on cancer treatment modalities and timing of those treatments in relation to COVID-19 outcomes by use of registry data with complete follow-up.

A few limitations need to be acknowledged. Although Norway, Denmark and Iceland represent a population of 9 million adults, the demographics of these countries are fairly homogenous which may limit the generalisability of the results to other populations. Data from Sweden and Finland were not included in this Nordic comparison, due to that linked population-based data from these two countries were not available to our team at the time of analysis. Despite the large sample size, some of the comparisons were hampered by low power due to low rates of severe COVID-19 outcomes in the study populations in 2020. In particular, the numbers were sparse in the subset analyses (during the first and second pandemic waves by cancer types and treatment modalities), as well as in Iceland, where we were unable to perform the analysis of severe COVID-19 outcomes. We were also unable to assess specific impacts of combination therapies, e.g. chemotherapy combined with radiotherapy or immunotherapy. Hence, caution is warranted when interpreting separate therapeutic classes, as our study approach did not allow for the evaluation of independent treatment effects. Cancer patients are a heterogeneous group consisting of recently diagnosed individuals and long-term survivors. We used two definitions, focusing on recently diagnosed patients (within one year) and less recently diagnosed (within five years). We realise that these definitions exclude long-term survivors, but we aimed to capture patients receiving oncologic treatment, either recently of currently. We did not have information on cancer stage, comorbidities, or body mass index, all of which are likely substantial confounders. In addition, no information on treatments for COVID-19 was available. Due to the possibility of false positive SARS-CoV-2 tests, the rates of COVID-19 outcomes may have been over-estimated leading to potential attenuation of the associations. While the Nordic countries have similar demographics, healthcare systems and population registries, differences exist in reporting of data and comparability of the health registries across the countries. Although mostly similar, there were some differences in pandemic preparedness and response measures in the Nordic region during the first year of the pandemic, which may have influenced our results.^30^ Testing activity differed in the Nordic countries during the first pandemic wave from March to May 2020, with limited testing availability prioritised to hospital patients and staff in Denmark and Norway, whereas access to diagnostic testing was more widely available in Iceland. Due to the limited testing availability in Denmark and Norway, we cannot exclude that differential testing bias could be present. During Summer 2020, testing became widely available across the populations with no restrictions.

## Conclusion

In the pre-vaccination era of the COVID-19 pandemic in 2020, cancer patients, in comparison to the general population, were at increased risk of developing severe COVID-19 outcomes including hospitalisation, intensive care admission and death. Although absolute risks were low, the relative risks of severe COVID-19 outcomes were particularly high in recently diagnosed and treated patients, including those treated with chemotherapy, immunotherapy and targeted therapy. The relative risks were also consistently increased in patients below age 70 years and patients with haematologic malignancies. Still, cancer patients were generally not at a higher risk of testing positive for SARS-CoV-2 compared to general population, with the exception of the first months of the pandemic when testing capacity was limited. While the findings were mostly consistent across the three countries, some analyses were hampered by few outcome events, particularly in Iceland and Norway. In the post-vaccination era, studies are warranted on the impact of COVID-19 treatments and vaccinations on the risk of severe outcomes in cancer patients.

## Contributors

ALVJ and GU conceived and designed the study. AS, TBJ, CWS, LSM, HB, and EJO compiled the national data from their respective countries. TBJ, SI, and SF harmonised the cancer treatment codes across countries. ALVJ and AS analysed the Norwegian data, ALVJ and CWS analysed the Danish data, and ALVJ and EJO analysed the Icelandic data. All authors interpreted the data and results. ALVJ and GU wrote the first draft of the paper. All authors critically reviewed and edited the manuscript. All authors approved the final manuscript. The work reported in the article has been performed by the authors, unless clearly specified in the text.

## Data sharing statement

This study was based on national health registry data. The data are available from the registry holder of each specific health registry under the use of appropriate ethical and legal permissions, including the GDPR. Further details and other data that support the findings of this study are available from the corresponding author upon request.

## Ethical statement

Denmark: Legal approval to use data was obtained from The Danish Health Data Authority. Ethical review is not required for registry-based studies in Denmark.

Norway: The Norwegian Cancer Registry provided statistical data for this study. The CRN has permission to collect, process and report statistical data without the need to seek consent. This project also has an ethical permission in Norway (REK 136 767).

Iceland: The Icelandic Cancer Registry (ICR) provided statistical data for this study. The ICR has permission to collect, process and report statistical data without the need to seek consent. This project has an ethical permission in Iceland (VSNb202 l 020023/03.0 I).

## Declaration of interests

ML declares stock or stock options in Astra Zeneca and Pfizer Inc. The other authors declare no competing interests.

## Appendix A Supplementary data

Supplementary Figures S1 and S2 and Tables S1–S5

STROBE-checklist
