# Supplementary material for: Were cancer patients worse off than the general population during the COVID-19 pandemic? A population-based study from Norway, Denmark and Iceland during the pre-vaccination era
Source: Lancet Reg Health Eur. 2023 Jul 10;31:100680. doi: 10.1016/j.lanepe.2023.100680 (PMC10398597; doi:10.1016/j.lanepe.2023.100680)
Supplement: Supplementary Figures S1 and S2 and Tables S1–S5 [file mmc1.pdf]

Supplementary material

Supplementary Table 1: Included data sources and health registries per country 2

Supplementary Table 2: Included COVID-19 and cancer diagnosis codes and treatment codes in each country 3

Supplementary Table 3: SIRs for SARS CoV-2 positive test for cancer patients (Norway, Denmark, Iceland) 4

Supplementary Table 4: SIRs for COVID-19 severe outcomes (hospitalization, intensive care, death) (Norway) 6

Supplementary Table 5: SIRs for COVID-19 severe outcomes (hospitalization, intensive care, death) (Denmark) 8

Supplementary Figure 1: Incidence of COVID-19 in Norway, Denmark, and Iceland per month during 2020 10

Supplementary Figure 2: Comparison of three estimation methods (SIR, OR and RR) (Norway) 11

Supplementary Table 1. Included health registries from each country.

	**Denmark**	**Norway**	**Iceland**
**Cancer registry (cancer diagnoses, treatments)**	Danish Cancer Registry<br><br>Danish National Patient Registry (treatments)	Cancer Registry of Norway<br><br>Norwegian National Patient Registry (treatments)<br><br>Norwegian Prescription Database (treatments)	Icelandic Cancer Registry
**Source of SARS-CoV2 and COVID-19 information**	MiBA, The Danish Microbiology Database for infectious diseases (Statens Seruminstitut)	MSIS, Norwegian Surveillance System for Communicable Diseases (confirmed positive)	COVID-19 Outpatient Clinic Registry at Landspitali–The National University Hospital of Iceland
**Patient Registry**	Danish National Patient Registry	Norwegian National Patient Registry	
**Intensive Care**	MiBA, The Danish Microbiology Database for infectious diseases (Statens Seruminstitut)	Norwegian Pandemic Registry (which is a part of the Norwegian Intensive care and pandemic registry)	
**Death Registry**	Cause of Death Registry	Cause of Death Registry	

Supplementary Table 2. Included codes and definitions of COVID-19-related outcomes, cancer diagnoses and treatments.

**COVID-19 outcomes**	
SARS CoV-2 positive	Confirmed positive SARS CoV-2 laboratory test (recorded in COVID-19 Registry)
COVID-19 hospitalization	a) hospital admission with primary diagnosis of laboratory confirmed COVID-19 (ICD-10 diagnosis code U07.1) in Patient Registry listed as discharge diagnosis, or<br>b) hospital admission in Patient Registry with previous positive COVID-19 confirmed laboratory test within 14 days in COVID-19 Registry
COVID-19 intensive care (ICU) admission	Norway:<br>Admission to intensive care with a COVID-19 diagnosis (U07.1) (primary diagnosis at discharge) in Patient Registry/Intensive Care Registry
	Denmark:<br>Admission to intensive care based on MiBA database. Procedure codes: NABB, NABE, BGDA0, BGDA1, BJFD0, BGXA2, BFHC92, BFHC93 og BFHC95
COVID-19 mechanical ventilation	Norway:<br>a) ventilator with intubation and invasive positive pressure ventilation
	Denmark:<br>a) ventilator with intubation and invasive positive pressure ventilation, or<br>b) other breathing support, including non-invasive positive pressure ventilation
COVID-19-related death	a) death with COVID-19 as underlying cause of death (U07.1) in Cause of Death Registry, or<br>b) death within 30 days after admission to hospital with COVID-19 as primary diagnosis (U07.1) in Patient Registry/Intensive Care Registry
**Cancer sites**	
All sites	ICD-10: C00-C96 (except C44), D45-D47, D32, D33, D42, D43, D35.3, D35.4, D44.3, D44.4, D44.5
Breast	ICD-10: C50
Prostate	ICD-10: C61
Melanoma	ICD-10: C43
Colorectal	ICD-10: C18-C20
Lung	ICD-10: C33-C34
Haematologic malignancy	ICD-10: C81-C96, D45-D47
**Cancer treatments**	
Chemotherapy	Norway:<br>ATC codes starting with: L01A, L01B, L01C, L01D, L01XA, L01XB, L01XX, L01XY in Prescription Drug Registry<br>Procedure codes: WBOC, JAQ10 in Patient Registry
	Denmark:<br>DRG codes starting with: BWHA, BOHJ1, BOHJ3 in Patient Registry
Radiotherapy	Norway:<br>Procedure codes: AAG50, AAG60, WEOA00, WEOA05, WEOA10, WEOA15, WEOB00, WEOB05, WEOB10, WEOB15, WEOB20, WEOC00, WEOC10 in Patient Registry<br>Radiotherapy treatment registered in the Cancer Registry
	Denmark:<br>DRG codes starting with: BWG in Patient Registry
Immunotherapy	Norway:<br>ATC codes starting with: L01XC11, L01XC17, L01XC18, L01XC32, L01XC28, L01XC31 in Prescription Drug Registry
	Denmark:<br>DRG codes starting with: BOHJ2, BWHB in Patient Registry
Targeted therapy	Norway:<br>ATC codes starting with L01XE, L01XC excluding immunotherapy codes (L01XD, L01XF, L01XG, L01XH, L01XJ, L01XK)
	Denmark:<br>DRG codes starting with: BWHC in Patient Registry

Supplementary Table 3. Standardised incidence ratios (SIRs) of SARS-CoV-2-positive test in cancer patients diagnosed within 1 or 5 years from the infection by subgroups of sex, months, age, cancer therapy and cancer types during March-December 2020 in Norway, Denmark and Iceland.

<table>
<tr><th></th><th colspan="2">Norway</th><th colspan="2">Denmark</th><th colspan="2">Iceland</th></tr>
<tr><th></th><th>Obs/Exp
N/N</th><th>SIR [95% CI]</th><th>Obs/Exp
N/N</th><th>SIR [95% CI]</th><th>Obs/Exp
N/N</th><th>SIR [95% CI]</th></tr>
<tr><td><u>Cancer within 1 year</u></td><td></td><td></td><td></td><td></td><td></td><td></td></tr>
<tr><td>All</td><td>203 / 192</td><td>1.06 [0.92-1.21]</td><td>727 / 679</td><td>1.07 [0.99-1.15]</td><td>19 / 19</td><td>1.02 [0.62-1.60]</td></tr>
<tr><td>Men</td><td>118 / 103</td><td>1.14 [0.95-1.37]</td><td>366 / 327</td><td>1.12 [1.01-1.24]</td><td>11 / 9</td><td>1.17 [0.59-2.10]</td></tr>
<tr><td>Women</td><td>85 / 89</td><td>0.96 [0.76-1.18]</td><td>361 / 352</td><td>1.03 [0.92-1.14]</td><td>8 / 9</td><td>0.87 [0.38-1.72]</td></tr>
<tr><td>Month 2020</td><td></td><td></td><td></td><td></td><td></td><td></td></tr>
<tr><td>Mar-Apr</td><td>65 / 48</td><td>1.37 [1.06-1.74]</td><td>98 / 72</td><td>1.36 [1.10-1.66]</td><td>5 / 7</td><td>0.73 [0.24-1.71]</td></tr>
<tr><td>May-Jun</td><td><5 / 4</td><td>1.06 [0.29-2.70]</td><td>37 / 17</td><td>2.12 [1.49-2.92]</td><td>- / -</td><td>N/A</td></tr>
<tr><td>Jul-Aug</td><td><5 / 5</td><td>0.40 [0.05-1.45]</td><td>15 / 14</td><td>1.05 [0.59-1.73]</td><td>1 / 1</td><td>0.99 [0.03-5.50]</td></tr>
<tr><td>Sep-Oct</td><td>23 / 30</td><td>0.78 [0.49-1.16]</td><td>100 / 113</td><td>0.88 [0.72-1.07]</td><td>10 / 8</td><td>1.29 [0.62-2.37]</td></tr>
<tr><td>Nov-Dec</td><td>203 / 192</td><td>1.06 [0.92-1.21]</td><td>477 / 461</td><td>1.03 [0.94-1.13]</td><td>3 / 3</td><td>1.06 [0.22-3.10]</td></tr>
<tr><td>Age</td><td></td><td></td><td></td><td></td><td></td><td></td></tr>
<tr><td>18-39</td><td>13 / 16</td><td>0.83 [0.44-1.43]</td><td>51 / 50</td><td>1.03 [0.76-1.35]</td><td rowspan="7">10 / 12</td><td rowspan="7">0.81 [0.39-1.49]</td></tr>
<tr><td>40-44</td><td><5 / 7</td><td>0.54 [0.15-1.38]</td><td>24 / 27</td><td>0.88 [0.57-1.31]</td></tr>
<tr><td>45-49</td><td>8 / 12</td><td>0.67 [0.29-1.32]</td><td>46 / 43</td><td>1.06 [0.78-1.42]</td></tr>
<tr><td>50-54</td><td>17 / 17</td><td>1.01 [0.59-1.61]</td><td>64 / 64</td><td>0.99 [0.76-1.27]</td></tr>
<tr><td>55-59</td><td>25 / 21</td><td>1.18 [0.76-1.74]</td><td>74 / 80</td><td>0.92 [0.73-1.16]</td></tr>
<tr><td>60-64</td><td>21 / 23</td><td>0.92 [0.57-1.41]</td><td>69 / 82</td><td>0.84 [0.65-1.06]</td></tr>
<tr><td>65-69</td><td>29 / 23</td><td>1.27 [0.85-1.83]</td><td>93 / 77</td><td>1.20 [0.97-1.47]</td></tr>
<tr><td>70-74</td><td>30 / 22</td><td>1.34 [0.91-1.92]</td><td>110 / 80</td><td>1.37 [1.13-1.66]</td><td rowspan="4">9 / 6</td><td rowspan="4">1.44 [0.66-2.74]</td></tr>
<tr><td>75-79</td><td>20 / 21</td><td>0.96 [0.58-1.47]</td><td>95 / 78</td><td>1.22 [0.98-1.49]</td></tr>
<tr><td>80-84</td><td>17 / 15</td><td>1.16 [0.68-1.86]</td><td>63 / 51</td><td>1.24 [0.95-1.58]</td></tr>
<tr><td>85+</td><td>19 / 16</td><td>1.22 [0.73-1.90]</td><td>38 / 45</td><td>0.84 [0.60-1.16]</td></tr>
<tr><td>Treatment within 3 months</td><td></td><td></td><td></td><td></td><td></td><td></td></tr>
<tr><td>Any treatment</td><td>68 / 60</td><td>1.13 [0.88-1.44]</td><td>242 / 215</td><td>1.12 [0.99-1.28]</td><td></td><td></td></tr>
<tr><td>CT any</td><td>56 / 47</td><td>1.19 [0.90-1.55]</td><td>176 / 157</td><td>1.12 [0.96-1.30]</td><td></td><td></td></tr>
<tr><td>CT only</td><td>35 / 31</td><td>1.14 [0.79-1.58]</td><td>167 / 147</td><td>1.13 [0.97-1.32]</td><td></td><td></td></tr>
<tr><td>RT any</td><td>24 / 20</td><td>1.17 [0.75-1.74]</td><td>77 / 70</td><td>1.09 [0.86-1.37]</td><td></td><td></td></tr>
<tr><td>Immunotherapy</td><td><5 / 3</td><td>0.93 [0.19-2.73]</td><td>51 / 38</td><td>1.35 [1.01-1.78]</td><td></td><td></td></tr>
<tr><td>Targeted therapy</td><td>9 / 7</td><td>1.36 [0.62-2.58]</td><td>79 / 63</td><td>1.26 [0.99-1.57]</td><td></td><td></td></tr>
<tr><td>Major cancer site</td><td></td><td></td><td></td><td></td><td></td><td></td></tr>
<tr><td>Breast</td><td>28 / 25</td><td>1.10 [0.73-1.60]</td><td>100 / 103</td><td>0.97 [0.79-1.18]</td><td></td><td></td></tr>
<tr><td>Prostate</td><td>35 / 30</td><td>1.18 [0.82-1.65]</td><td>76 / 76</td><td>1.01 [0.79-1.26]</td><td></td><td></td></tr>
<tr><td>Melanoma</td><td>21 / 18</td><td>1.19 [0.74-1.82]</td><td>59 / 61</td><td>0.96 [0.73-1.24]</td><td></td><td></td></tr>
<tr><td>Colorectal</td><td>24 / 25</td><td>0.97 [0.62-1.44]</td><td>92 / 75</td><td>1.22 [0.99-1.50]</td><td></td><td></td></tr>
<tr><td>Lung</td><td>11 / 15</td><td>0.72 [0.36-1.29]</td><td>63 / 61</td><td>1.04 [0.80-1.33]</td><td></td><td></td></tr>
<tr><td>Haematologic</td><td>28 / 19</td><td>1.51 [1.00-2.18]</td><td>123 / 99</td><td>1.25 [1.04-1.49]</td><td></td><td></td></tr>
<tr><td></td><td></td><td></td><td></td><td></td><td></td><td></td></tr>
<tr><td><u>Cancer within 5 years</u></td><td></td><td></td><td></td><td></td><td></td><td></td></tr>
<tr><td>All</td><td>730 / 765</td><td>0.95 [0.89-1.03]</td><td>2758 / 2704</td><td>1.02 [0.98-1.06]</td><td>72 / 72</td><td>1.00 [0.79-1.27]</td></tr>
<tr><td>Men</td><td>381 / 404</td><td>0.94 [0.85-1.04]</td><td>1343 / 1264</td><td>1.06 [1.01-1.12]</td><td>40 / 34</td><td>1.18 [0.84-1.61]</td></tr>
<tr><td>Women</td><td>349 / 361</td><td>0.97 [0.87-1.07]</td><td>1415 / 1440</td><td>0.98 [0.93-1.04]</td><td>32 / 38</td><td>0.85 [0.58-1.19]</td></tr>
</table>

<table>
<tr><td>Month 2020</td><td></td><td></td><td></td><td></td><td></td><td></td></tr>
<tr><td>Mar-Apr</td><td>217 / 193</td><td>1.13 [0.98-1.29]</td><td>303 / 275</td><td>1.10 [0.98-1.23]</td><td>28 / 26</td><td>1.07 [0.71-1.55]</td></tr>
<tr><td>May-Jun</td><td>11 / 15</td><td>0.71 [0.36-1.28]</td><td>92 / 68</td><td>1.35 [1.09-1.65]</td><td>- / -</td><td>N/A</td></tr>
<tr><td>Jul-Aug</td><td>14 / 20</td><td>0.70 [0.38-1.17]</td><td>46 / 56</td><td>0.82 [0.60-1.10]</td><td>2 / 4</td><td>0.51 [0.06-1.84]</td></tr>
<tr><td>Sep-Oct</td><td>111 / 117</td><td>0.95 [0.78-1.14]</td><td>446 / 448</td><td>1.00 [0.90-1.09]</td><td>33 / 30</td><td>1.10 [0.76-1.54]</td></tr>
<tr><td>Nov-Dec</td><td>377 / 420</td><td>0.90 [0.81-0.99]</td><td>1871 / 1857</td><td>1.01 [0.96-1.05]</td><td>9 / 11</td><td>0.81 [0.37-1.53]</td></tr>
<tr><td>Age at COVID-19 diagnosis (years)</td><td></td><td></td><td></td><td></td><td></td><td></td></tr>
<tr><td>18-39</td><td>58 / 62</td><td>0.94 [0.71-1.21]</td><td>175 / 192</td><td>0.91 [0.78-1.06]</td><td rowspan="7">46 / 48</td><td rowspan="7">0.95 [0.70-1.27]</td></tr>
<tr><td>40-44</td><td>24 / 31</td><td>0.76 [0.49-1.14]</td><td>125 / 111</td><td>1.13 [0.94-1.35]</td></tr>
<tr><td>45-49</td><td>39 / 48</td><td>0.82 [0.58-1.12]</td><td>168 / 177</td><td>0.95 [0.81-1.10]</td></tr>
<tr><td>50-54</td><td>70 / 68</td><td>1.03 [0.80-1.30]</td><td>266 / 265</td><td>1.00 [0.89-1.13]</td></tr>
<tr><td>55-59</td><td>78 / 84</td><td>0.93 [0.73-1.15]</td><td>315 / 318</td><td>0.99 [0.88-1.10]</td></tr>
<tr><td>60-64</td><td>82 / 89</td><td>0.92 [0.73-1.14]</td><td>303 / 322</td><td>0.94 [0.84-1.05]</td></tr>
<tr><td>65-69</td><td>89 / 88</td><td>1.01 [0.81-1.24]</td><td>312 / 296</td><td>1.05 [0.94-1.18]</td></tr>
<tr><td>70-74</td><td>85 / 89</td><td>0.95 [0.76-1.18]</td><td>383 / 328</td><td>1.17 [1.05-1.29]</td><td rowspan="4">26 / 23</td><td rowspan="4">1.11 [0.73-1.63]</td></tr>
<tr><td>75-79</td><td>77 / 82</td><td>0.94 [0.74-1.17]</td><td>324 / 309</td><td>1.05 [0.94-1.17]</td></tr>
<tr><td>80-84</td><td>59 / 59</td><td>1.00 [0.76-1.30]</td><td>202 / 196</td><td>1.03 [0.90-1.19]</td></tr>
<tr><td>85+</td><td>69 / 64</td><td>1.08 [0.84-1.36]</td><td>185 / 191</td><td>0.97 [0.83-1.12]</td></tr>
<tr><td>Treatment within 3 months</td><td></td><td></td><td></td><td></td><td></td><td></td></tr>
<tr><td>Any treatment</td><td>136 / 130</td><td>1.05 [0.88-1.24]</td><td>537 / 471</td><td>1.14 [1.05-1.24]</td><td></td><td></td></tr>
<tr><td>CT any</td><td>111 / 104</td><td>1.07 [0.88-1.29]</td><td>363 / 303</td><td>1.20 [1.08-1.33]</td><td></td><td></td></tr>
<tr><td>CT only</td><td>77 / 74</td><td>1.04 [0.82-1.30]</td><td>349 / 287</td><td>1.21 [1.09-1.35]</td><td></td><td></td></tr>
<tr><td>RT any</td><td>38 / 29</td><td>1.30 [0.92-1.78]</td><td>106 / 90</td><td>1.18 [0.97-1.43]</td><td></td><td></td></tr>
<tr><td>Immunotherapy</td><td>8 / 7</td><td>1.11 [0.48-2.19]</td><td>186 / 164</td><td>1.14 [0.98-1.31]</td><td></td><td></td></tr>
<tr><td>Targeted therapy</td><td>20 / 22</td><td>0.92 [0.56-1.42]</td><td>211 / 162</td><td>1.30 [1.13-1.49]</td><td></td><td></td></tr>
<tr><td>Major cancer site</td><td></td><td></td><td></td><td></td><td></td><td></td></tr>
<tr><td>Breast</td><td>113 / 116</td><td>0.97 [0.80-1.17]</td><td>455 / 480</td><td>0.95 [0.86-1.04]</td><td></td><td></td></tr>
<tr><td>Prostate</td><td>122 / 136</td><td>0.90 [0.74-1.07]</td><td>347 / 340</td><td>1.02 [0.92-1.13]</td><td></td><td></td></tr>
<tr><td>Melanoma</td><td>84 / 76</td><td>1.10 [0.88-1.36]</td><td>304 / 280</td><td>1.09 [0.97-1.22]</td><td></td><td></td></tr>
<tr><td>Colorectal</td><td>94 / 100</td><td>0.94 [0.76-1.15]</td><td>309 / 327</td><td>0.94 [0.84-1.06]</td><td></td><td></td></tr>
<tr><td>Lung</td><td>28 / 41</td><td>0.69 [0.46-1.00]</td><td>152 / 168</td><td>0.91 [0.77-1.06]</td><td></td><td></td></tr>
<tr><td>Haematologic</td><td>98 / 77</td><td>1.27 [1.03-1.55]</td><td>480 / 397</td><td>1.21 [1.10-1.32]</td><td></td><td></td></tr>
</table>

CT: chemotherapy; RT: radiotherapy.

Supplementary Table 4. Standardised incidence ratios (SIRs) of COVID-19-related hospitalization, intensive care (ICU) admission, mechanical ventilation and death in cancer patients diagnosed within 1 or 5 years from the admission or death by subgroups of sex, age and cancer therapy during March-December 2020 in Norway.

	**COVID-19 Hospitalisation**	**COVID-19 Hospitalisation**	**COVID-19 ICU admission**	**COVID-19 ICU admission**	**COVID-19 Mechanical ventilation**	**COVID-19 Mechanical ventilation**	**COVID-19 Death**	**COVID-19 Death**
	**Obs/Exp N/N**	**SIR [95% CI]**	**Obs/Exp N/N**	**SIR [95% CI]**	**Obs/Exp N/N**	**SIR [95% CI]**	**Obs/Exp N/N**	**SIR [95% CI]**
**Cancer within 1 year**								
All	67 / 28	2.43 [1.89-3.09]	17 / 5	3.19 [1.86-5.11]	8 / 4	1.94 [0.84-3.82]	18 / 8	2.18 [1.29-3.45]
Men	42 / 18	2.36 [1.70-3.20]	13 / 4	3.19 [1.70-5.46]	7 / 3	2.21 [0.89-4.55]	12 / 5	2.31 [1.19-4.03]
Women	25 / 10	2.56 [1.66-3.78]	<5 / 1	3.20 [0.87-8.18]	<5 / 1	1.04 [0.03-5.79]	6 / 3	1.97 [0.72-4.29]
**Month 2020**								
Mar-Apr	26 / 13	2.04 [1.34-2.99]	6 / 3	2.24 [0.82-4.87]	<5 / 2	1.69 [0.46-4.33]	7 / 4	1.75 [0.70-3.61]
May-Jun	<5 / 1	1.99 [0.24-7.19]	<5 / 0	3.81 [0.10-21.23]	-/-	-	<5 / 1	5.58 [1.15-16.31]
Jul-Aug	-/-	-	-/-	-	-/-	-	-/-	-
Sep-Oct	11 / 4	3.02 [1.51-5.40]	<5 / 1	4.46 [0.92-13.04]	<5 / 0	2.20 [0.06-12.24]	<5 / 0	3.17 [0.08-17.68]
Nov-Dec	28 / 9	3.00 [1.99-4.34]	7 / 2	4.45 [1.79-9.17]	<5 / 1	2.71 [0.56-7.91]	7 / 3	2.12 [0.85-4.38]
**Age (years)**								
18-69	27 / 10	2.84 [1.87-4.13]	9 / 2	4.39 [2.01-8.32]	<5 / 2	2.40 [0.65-6.14]	5 / 1	7.71 [2.50-18.00]
70+	40 / 18	2.22 [1.59-3.02]	8 / 3	2.44 [1.05-4.81]	<5 / 2	1.63 [0.44-4.16]	13 / 8	1.71 [0.91-2.92]
**Treatment within 3 months**								
Any treatment	31 / 8	4.02 [2.73-5.71]	6 / 2	3.93 [1.44-8.56]	<5 / 1	2.53 [0.52-7.38]	<5 / 2	2.30 [0.63-5.89]
CT any	23 / 6	4.10 [2.60-6.15]	6 / 1	5.35 [1.96-11.64]	<5 / 1	3.42 [0.71-10.01]	<5 / 1	2.74 [0.56-8.00]
CT only	18 / 4	4.75 [2.81-7.50]	5 / 1	6.61 [2.15-15.42]	<5 / 1	5.09 [1.05-14.88]	<5 / 1	2.49 [0.30-9.01]
RT any	9 / 3	3.27 [1.49-6.21]	<5 / 1	1.86 [0.05-10.36]	-/-	N/A	<5 / 1	1.50 [0.04-8.38]
**Major sites**								
Breast	6 / 2	2.49 [0.91-5.41]	-/-	N/A	-/-	N/A	<5 / 1	3.29 [0.40-11.87]
Prostate	12 / 6	2.16 [1.11-3.77]	<5 / 1	2.18 [0.45-6.37]	<5 / 1	2.78 [0.57-8.13]	<5 / 1	0.69 [0.02-3.87]
Melanoma	4 / 2	1.73 [0.47-4.44]	-/-	N/A	-/-	N/A	-/-	N/A
Colorectal	8 / 4	1.93 [0.83-3.80]	<5 / 1	4.03 [0.83-11.79]	<5 / 1	1.76 [0.04-9.79]	<5 / 2	0.64 [0.02-3.58]
Lung	5 / 3	1.94 [0.63-4.53]	-/-	N/A	-/-	N/A	<5 / 1	4.07 [0.84-11.90]
Haematologic	12 / 3	4.37 [2.26-7.63]	<5 / 1	7.64 [2.08-19.56]	<5 / 0	7.39 [1.52-21.59]	5 / 1	5.64 [1.83-13.16]
**Cancer within 5 years**								
All	174 / 110	1.58 [1.35-1.83]	38 / 21	1.80 [1.28-2.47]	28 / 16	1.71 [1.14-2.47]	40 / 34	1.17 [0.84-1.60]
Men	100 / 70	1.42 [1.15-1.73]	26 / 16	1.63 [1.06-2.38]	19 / 12	1.53 [0.92-2.39]	21 / 21	0.98 [0.61-1.49]
Women	74 / 40	1.87 [1.47-2.34]	12 / 5	2.36 [1.22-4.12]	9 / 4	2.28 [1.04-4.33]	19 / 13	1.51 [0.91-2.36]
**Month 2020**								
Mar-Apr	75 / 52	1.44 [1.13-1.81]	17 / 11	1.58 [0.92-2.54]	15 / 9	1.59 [0.89-2.62]	15 / 17	0.87 [0.49-1.43]
May-Jun	<5 / 4	0.98 [0.27-2.50]	<5 / 1	1.88 [0.23-6.79]	<5 / 1	1.56 [0.04-8.72]	<5 / 2	1.29 [0.27-3.76]
Jul-Aug	<5 / 3	0.90 [0.19-2.64]	- / -	-	- / -	-	<5 / 0	4.58 [0.55-16.53]
Sep-Oct	28 / 14	1.95 [1.30-2.82]	7 / 3	2.65 [1.07-5.46]	<5 / 2	2.23 [0.61-5.72]	<5 / 1	0.80 [0.02-4.47]
Nov-Dec	64 / 36	1.76 [1.36-2.25]	12 / 6	1.97 [1.02-3.44]	8 / 4	1.86 [0.80-3.66]	19 / 13	1.49 [0.90-2.33]

**Age**								
18-69	59 / 37	1.59 [1.21-2.05]	17 / 8	2.15 [1.25-3.44]	12 / 6	1.87 [0.96-3.26]	9 / 3	3.59 [1.64-6.82]
70+	115 / 73	1.57 [1.30-1.89]	21 / 13	1.59 [0.99-2.43]	16 / 10	1.61 [0.92-2.61]	31 / 32	0.98 [0.67-1.40]
**Treatment within 3 months**								
Any treatment	52 / 17	3.04 [2.27-3.98]	9 / 3	2.73 [1.25-5.19]	6 / 3	2.34 [0.86-5.10]	9 / 4	2.23 [1.02-4.23]
CT any	41 / 13	3.15 [2.26-4.27]	9 / 3	3.59 [1.64-6.81]	6 / 2	3.06 [1.12-6.67]	8 / 3	2.93 [1.27-5.78]
CT only	30 / 10	3.14 [2.12-4.49]	7 / 2	3.88 [1.56-7.99]	5 / 1	3.55 [1.15-8.29]	5 / 2	2.35 [0.76-5.47]
RT any	13 / 4	3.19 [1.70-5.46]	<5 / 1	1.24 [0.03-6.88]	-/-	-	<5 / 1	1.97 [0.24-7.11]
**Major sites**								
Breast	18 / 12	1.57 [0.93-2.47]	-/-	-	-/-	-	<5 / 3	1.35 [0.37-3.46]
Prostate	36 / 27	1.31 [0.92-1.81]	7 / 7	1.06 [0.43-2.18]	7 / 5	1.35 [0.54-2.79]	<5 / 8	0.50 [0.14-1.29]
Melanoma	11 / 10	1.09 [0.55-1.96]	-/-	-	-/-	-	-/-	-
Colorectal	24 / 17	1.39 [0.89-2.06]	<5 / 3	1.31 [0.36-3.36]	<5 / 2	0.86 [0.10-3.09]	<5 / 7	0.43 [0.09-1.26]
Lung	11 / 7	1.56 [0.78-2.79]	-/-	-	-/-	-	5 / 2	2.43 [0.79-5.66]
Haematologic	32 / 11	2.87 [1.96-4.05]	11 / 2	5.18 [2.59-9.27]	10 / 2	6.09 [2.92-11.20]	13 / 4	3.67 [1.95-6.28]

CT: chemotherapy; RT: radiotherapy.

Supplementary Table 5. Standardised incidence ratios (SIRs) of COVID-19-related hospitalization, intensive care (ICU) admission, mechanical ventilation and death in cancer patients diagnosed within 1 or 5 years from the admission or death by subgroups of sex, age and cancer therapy. March-December 2020 in Denmark.

	COVID-19 Hospitalisation	COVID-19 Hospitalisation	COVID-19 ICU admission	COVID-19 ICU admission	COVID-19 Mechanical ventilation	COVID-19 Mechanical ventilation	COVID-19 Death	COVID-19 Death
	Obs/Exp N/N	SIR [95% CI]	Obs/Exp N/N	SIR [95% CI]	Obs/Exp N/N	SIR [95% CI]	Obs/Exp N/N	SIR [95% CI]
**Cancer within 1 year**								
All	241 / 108	2.23 [1.96-2.54]	35 / 14	2.52 [1.75-3.50]	27 / 9	3.02 [1.99-4.39]	44 / 19	2.29 [1.66-3.07]
Men	146 / 67	2.18 [1.84-2.56]	25 / 10	2.43 [1.57-3.58]	18 / 7	2.72 [1.61-4.30]	30 / 13	2.30 [1.55-3.28]
Women	95 / 41	2.32 [1.88-2.84]	10 / 4	2.77 [1.33-5.10]	9 / 2	3.88 [1.77-7.36]	14 / 6	2.26 [1.24-3.79]
**Month 2020**								
Mar-Apr	60 / 32	1.87 [1.42-2.40]	12 / 5	2.23 [1.15-3.90]	10 / 4	2.47 [1.19-4.54]	10 / 8	1.29 [0.62-2.38]
May-Jun	36 / 6	5.70 [3.99-7.89]	7 / 1	10.75 [4.32-22.15]	7 / 1	11.51 [4.63-23.71]	11 / 2	4.91 [2.45-8.78]
Jul-Aug	5 / 2	2.40 [0.78-5.59]	<5 / 0	**N/A**	<5 / 0	N/A	<5 / 0	2.33 [0.06-12.97]
Sep-Oct	23 / 12	1.89 [1.20-2.84]	<5 / 1	1.44 [0.17-5.19]	<5 / 1	1.37 [0.03-7.64]	<5 / 1	3.00 [0.82-7.69]
Nov-Dec	117 / 55	2.12 [1.76-2.54]	14 / 6	2.22 [1.22-3.73]	9 / 3	2.58 [1.18-4.90]	18 / 8	2.39 [1.42-3.78]
**Age (years)**								
18-69	108 / 29	3.70 [3.03-4.46]	17 / 4	4.11 [2.40-6.59]	15 / 3	5.24 [2.93-8.64]	8 / 1	5.40 [2.33-10.64]
70+	133 / 79	1.69 [1.42-2.00]	18 / 10	1.84 [1.09-2.91]	12 / 6	1.97 [1.02-3.45]	36 / 18	2.03 [1.42-2.80]
**Treatment within 3 months**								
Any treatment	111 / 30	3.76 [3.09-4.53]	20 / 4	5.00 [3.05-7.72]	16 / 3	6.15 [3.51-9.99]	26 / 4	5.78 [3.78-8.47]
CT any	98 / 20	4.92 [3.99-5.99]	20 / 3	7.27 [4.44-11.24]	16 / 2	8.93 [5.10-14.50]	18 / 3	6.30 [3.73-9.96]
CT only	92 / 19	4.89 [3.94-5.99]	18 / 3	6.94 [4.11-10.97]	15 / 2	8.87 [4.97-14.64]	18 / 3	6.61 [3.92-10.44]
RT any	29 / 10	2.97 [1.99-4.26]	<5 / 1	2.26 [0.47-6.60]	<5 / 1	2.31 [0.28-8.36]	9 / 2	5.95 [2.72-11.30]
Immunotherapy	25 / 5	4.59 [2.97-6.78]	<5 / 1	5.79 [1.58-14.82]	5 / 0	11.18 [3.63-26.10]	7 / 1	8.26 [3.32-17.03]
Targeted therapy	50 / 9	5.70 [4.23-7.52]	11 / 1	9.55 [4.77-17.08]	9 / 1	12.09 [5.53-22.95]	8 / 1	5.73 [2.47-11.29]
**Major sites**								
Breast	16 / 11	1.46 [0.84-2.38]	<5 / 1	2.12 [0.26-7.66]	<5 / 1	3.24 [0.39-11.71]	<5 / 2	0.62 [0.02-3.44]
Prostate	21 / 18	1.18 [0.73-1.81]	<5 / 3	0.35 [0.01-1.94]	<5 / 2	0.54 [0.01-3.01]	<5 / 3	1.19 [0.33-3.06]
Melanoma	10 / 7	1.35 [0.65-2.49]	<5 / 1	3.33 [0.69-9.74]	<5 / 1	3.47 [0.42-12.55]	<5 / 1	0.79 [0.02-4.42]
Colorectal	32 / 15	2.13 [1.45-3.00]	7 / 2	3.87 [1.55-7.97]	<5 / 1	2.61 [0.54-7.63]	<5 / 3	1.30 [0.35-3.33]
Lung	26 / 12	2.25 [1.47-3.30]	<5 / 2	1.29 [0.16-4.65]	<5 / 1	1.00 [0.03-5.57]	11 / 2	5.42 [2.71-9.70]
Haematologic	73 / 18	3.96 [3.10-4.98]	14 / 2	5.93 [3.24-9.94]	13 / 2	8.57 [4.56-14.66]	15 / 4	4.15 [2.32-6.85]
**Cancer within 5 years**								
All	652 / 425	1.54 [1.42-1.66]	76 / 54	1.41 [1.11-1.76]	56 / 35	1.62 [1.22-2.10]	108 / 76	1.42 [1.17-1.72]
Men	393 / 259	1.52 [1.37-1.68]	53 / 40	1.34 [1.00-1.75]	37 / 25	1.46 [1.03-2.01]	69 / 51	1.36 [1.06-1.73]
Women	259 / 166	1.56 [1.38-1.76]	23 / 14	1.59 [1.01-2.39]	19 / 9	2.06 [1.24-3.22]	39 / 25	1.54 [1.10-2.11]
**Month 2020**								
Mar-Apr	174 / 122	1.43 [1.22-1.65]	33 / 20	1.63 [1.12-2.28]	27 / 15	1.77 [1.16-2.57]	32 / 30	1.08 [0.74-1.53]
May-Jun	58 / 25	2.36 [1.79-3.05]	9 / 3	3.58 [1.64-6.79]	10 / 2	4.25 [2.04-7.82]	19 / 9	2.14 [1.29-3.34]
Jul-Aug	15 / 8	1.83 [1.02-3.01]	<5 / 1	2.53 [0.31-9.13]	<5 / 0	3.76 [0.10-20.97]	<5 / 2	0.58 [0.01-3.25]
Sep-Oct	78 / 48	1.62 [1.28-2.02]	7 / 5	1.28 [0.52-2.64]	<5 / 3	0.70 [0.08-2.52]	11 / 5	2.07 [1.03-3.71]

Nov-Dec	327 / 221	1.48 [1.32-1.65]	25 / 25	1.00 [0.65-1.47]	16 / 14	1.16 [0.66-1.88]	45 / 31	1.48 [1.08-1.97]
**Age**								
18-69	234 / 112	2.08 [1.83-2.37]	26 / 16	1.66 [1.08-2.43]	20 / 11	1.85 [1.13-2.85]	21 / 6	3.77 [2.33-5.76]
70+	418 / 312	1.34 [1.21-1.47]	50 / 38	1.30 [0.97-1.72]	36 / 24	1.52 [1.06-2.10]	87 / 70	1.24 [0.99-1.53]
**Treatment within 3 months**								
Any treatment	208 / 67	3.09 [2.68-3.53]	26 / 9	2.93 [1.92-4.30]	22 / 6	3.85 [2.41-5.83]	45 / 11	4.25 [3.10-5.68]
CT any	166 / 43	3.89 [3.32-4.53]	26 / 6	4.55 [2.97-6.67]	22 / 4	5.97 [3.74-9.03]	28 / 7	4.25 [2.82-6.14]
CT only	159 / 40	3.93 [3.34-4.59]	23 / 5	4.25 [2.69-6.38]	20 / 3	5.72 [3.50-8.84]	28 / 6	4.46 [2.96-6.45]
RT any	44 / 13	3.39 [2.47-4.55]	<5 / 2	2.25 [0.61-5.77]	<5 / 1	2.60 [0.54-7.60]	14 / 2	6.80 [3.72-11.40]
Immunotherapy	65 / 23	2.85 [2.20-3.63]	8 / 3	2.78 [1.20-5.48]	10 / 2	5.43 [2.61-9.99]	18 / 3	5.15 [3.05-8.14]
Targeted therapy	93 / 24	3.93 [3.17-4.81]	15 / 3	4.86 [2.72-8.01]	13 / 2	6.56 [3.49-11.22]	16 / 4	4.17 [2.38-6.77]
**Major sites**								
Breast	60 / 51	1.17 [0.89-1.51]	7 / 4	1.57 [0.63-3.23]	7 / 3	2.42 [0.97-4.99]	10 / 8	1.32 [0.63-2.43]
Prostate	89 / 84	1.06 [0.85-1.31]	13 / 13	0.97 [0.52-1.66]	10 / 9	1.16 [0.56-2.14]	22 / 17	1.33 [0.83-2.01]
Melanoma	31 / 33	0.93 [0.63-1.32]	6 / 4	1.48 [0.54-3.23]	<5 / 3	1.55 [0.42-3.97]	<5 / 6	0.53 [0.11-1.56]
Colorectal	79 / 67	1.17 [0.93-1.46]	9 / 8	1.08 [0.49-2.04]	5 / 5	0.94 [0.31-2.20]	13 / 14	0.95 [0.51-1.63]
Lung	59 / 32	1.85 [1.41-2.38]	<5 / 4	0.71 [0.15-2.07]	<5 / 3	0.74 [0.09-2.67]	18 / 6	3.23 [1.91-5.10]
Haematologic	190 / 72	2.65 [2.29-3.06]	23 / 9	2.59 [1.64-3.88]	20 / 6	3.55 [2.17-5.48]	26 / 14	1.87 [1.22-2.74]

CT: chemotherapy; RT: radiotherapy.

Supplementary Figure 1. Incidence rates of SARS-CoV-2 infections in Norway, Denmark and Iceland during January-December 2020.

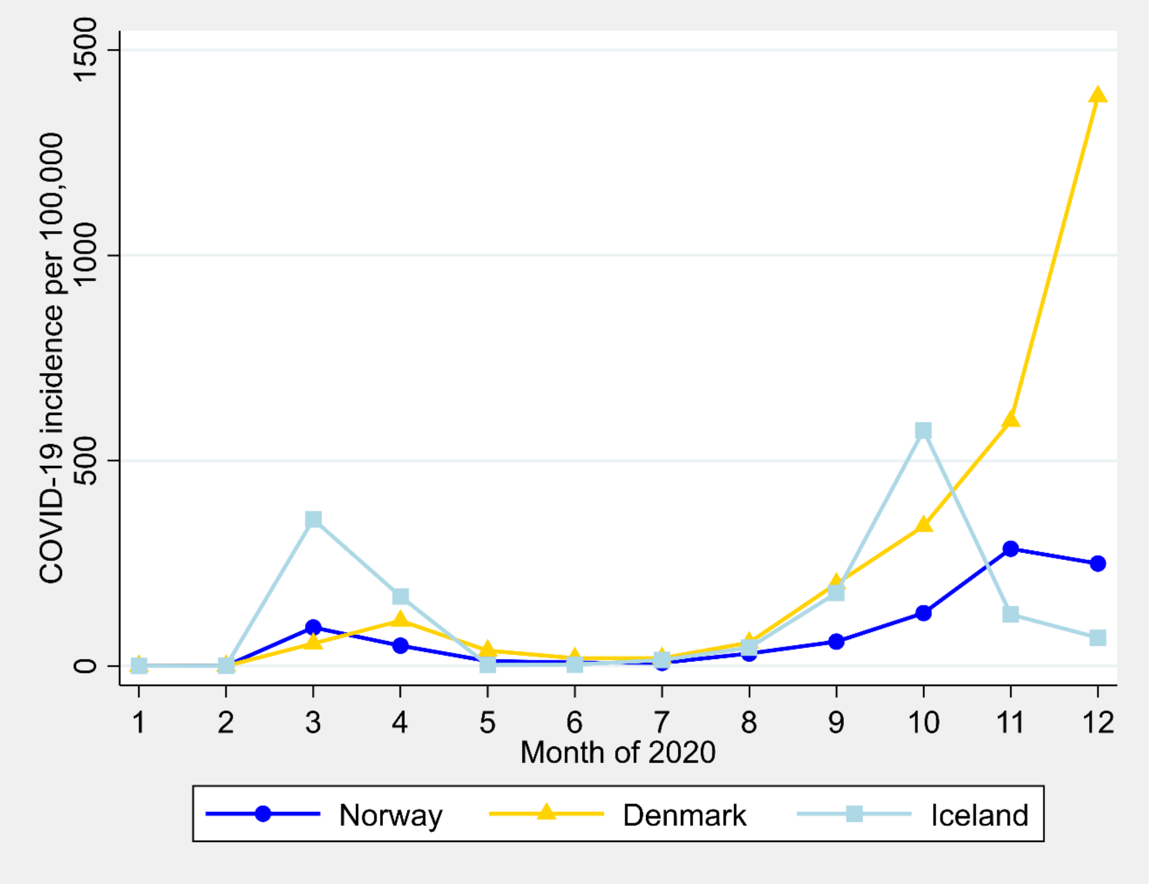

Johansson ALV, Laronningen S, Skovlund CW, et al. The impact of the COVID-19 pandemic on cancer diagnosis based on pathology notifications: A comparison across the Nordic countries during 2020. Int J Cancer 2022; 151(3):381-395.

Supplementary Figure 2. Comparison of three methods (SIR, OR from logistic regression and RR from Poisson regression) for estimating associations between cancer within 5 years (yes vs no) and severe COVID-19 outcomes. Norway only.

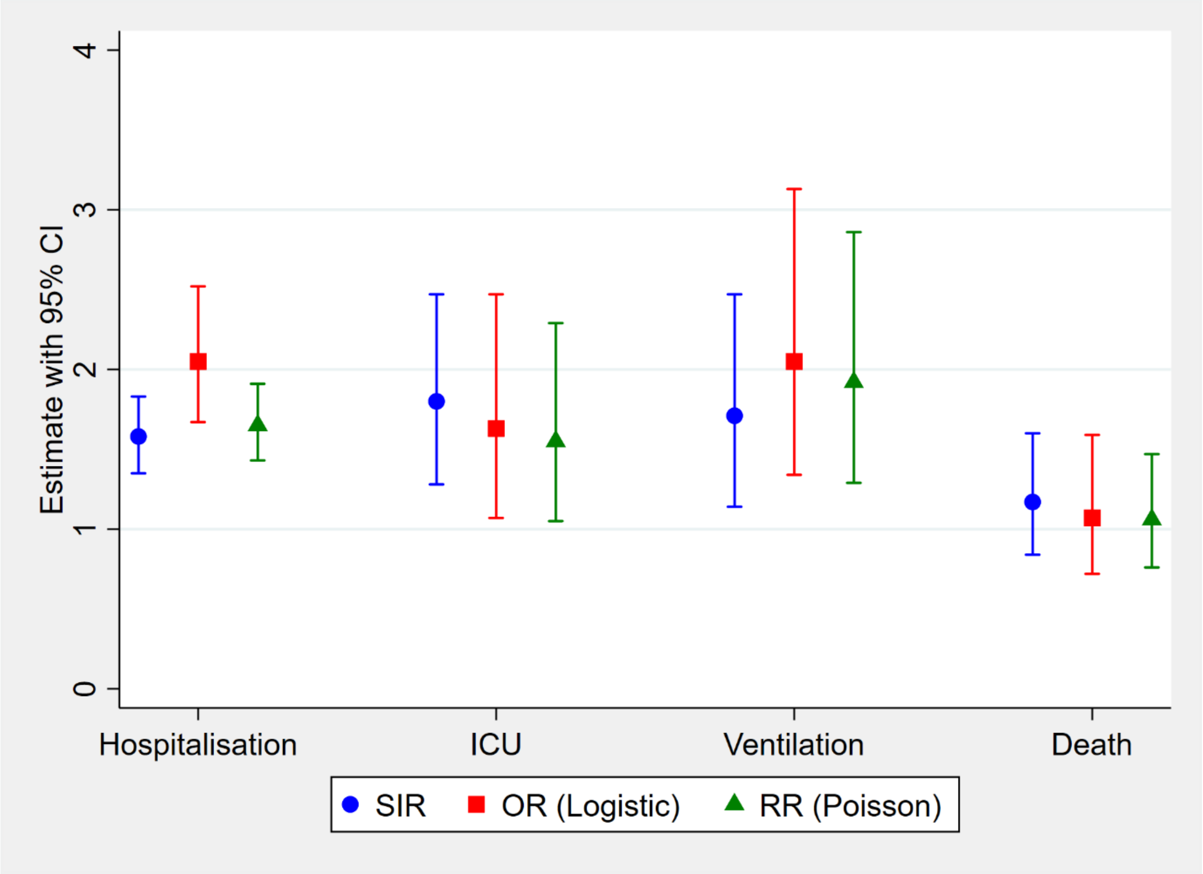

Logistic regression and Poisson regression models modelled COVID-19 outcomes (yes vs no) within 30 days of positive SARS-CoV-2 test by exposure status (cancer within 5 years: yes vs no), and with adjustment for age, sex, month, and region.
